# PES1 promotes the occurrence and development of papillary thyroid cancer by upregulating the ERα/ERβ protein ratio

**DOI:** 10.1038/s41598-018-37648-7

**Published:** 2019-01-31

**Authors:** Yi-Bo Qiu, Ling-Yao Liao, Rong Jiang, Man Xu, Lin-Wan Xu, George G. Chen, Zhi-Min Liu

**Affiliations:** 1 Department of Biochemistry and Molecular Biology, Molecular Medicine and Cancer Research Center, Chongqing Medical University, Chongqing, China; 2 Department of Pathology, Molecular Medicine and Cancer Research Center, Chongqing Medical University, Chongqing, China; 3 Department of Surgery, Chinese University of Hong Kong, Prince of Wales Hospital, Shatin, N.T., Hong Kong, China

## Abstract

PES1, a BRCT domain-containing protein, has been shown to play a role in modulating the balance and ratio between ERα and ERβ protein, which is involved in the occurrence and development of breast and ovarian cancer. However, its role in connection with the balance and ratio between ERα and ERβ protein in papillary thyroid cancer (PTC) remains unclear. Here, we found that ERα and ERβ were co-expressed in human PTC tissues and cells. ERα promoted and ERβ inhibited the proliferation, invasion and migration of PTC cells. PES1 modulated the balance between ERα and ERβ by elevating the ERα protein level and simultaneously reducing the ERβ protein level, then upregulating the ERα/ERβ protein ratio and promoting the proliferation, invasion and migration of PTC cells. In PTC tissues, PES1 protein level was positively correlated with the ERα protein level and negatively correlated with the ERβ protein level. The PES1 and ERα protein levels were gradually increased and the ERβ protein level was decreased by degree in the occurrence and development of PTC. Increased PES1 and ERα protein levels and decreased ERβ protein level were correlated with the aggressive behaviors of PTC patients such as large tumor size, extrathyroidal extension (ETE), lymph node metastasis (LNM), high BRAFV600E expression and high TNM stage. It is suggested that PES1 promotes the occurrence and development of PTC by elevating the ERα protein level and reducing the ERβ protein level, and then upregulating the ERα/ERβ protein ratio.

## Introduction

Papillary thyroid cancer (PTC) is three times more frequent in women than in men, with the greatest gender difference observed during reproductive years and the decreased incidence after menopause^1,2^. The elevated risk was also reported in women who used estrogen for gynecological problems and in women who used postmenopausal hormone replacement therapy or oral contraception^3–5^. It is suggested that estrogen may be involved in the occurrence and development of PTC, as has been shown in breast, endometrial and ovarian cancer^6^.

Estrogen exerts its physiological and pathophysiological actions largely through two estrogen receptors, ERα and ERβ, which belong to the steroid hormone receptor family^7,8^. ERα and ERβ are architecturally similar with three functional domains: N-terminal domain (NTD), DNA binding domain (DBD) and ligand binding domain (LBD). The two ERs share 97% similarity in their DBD and 59% in LBD, whereas the NTD is merely 16% similar^9^. The differences in their structures suggest that ERα and ERβ may have different functions. It is well known that ERα expression is associated with aberrant proliferation and the development of malignancy, in contrast, ERβ has been shown to inhibit cell proliferation, migration and invasion^10,11^. Although there is a controversy regarding the prognostic and predictive roles of ERβ expression, most of the studies that have analyzed a large number of samples have demonstrated a correlation of ERβ expression with a better clinical outcome in estrogen related cancer^12,13^. Lots of studies have shown that ERα promotes cell proliferation, invasion and migration and has been shown to have tumor-promoting effects, whereas ERβ may play an inhibitory role against the ERα-mediated tumor-promoting effects, especially when co-expressed with ERα^14–16^. The ERα/ERβ protein ratio would be critical in defining the overall response. Therefore, the imbalance between ERα and ERβ protein levels and the elevated ERα/ERβ protein ratio may be implicated in the occurrence and development of tumor in estrogen responsive organ^17,18^. Previous studies have shown that like the typical estrogen responsive organ such as breast, uterus and ovary, both ERα and ERβ are co-expressed in the normal and tumor tissues of the thyroid^19,20^. Moreover, like in breast, endometrial and ovarian cancer, ERα protein is increased, ERβ protein is decreased and finally the ERα/ERβ protein ratio is upregulated, which is involved in the occurrence and development of PTC^21–24^. However, how the protein levels of ERα and ERβ are modulated and how the ERα/ERβ protein ratio is upregulated in PTC remain unclear.

PES1, a breast cancer–associated gene 1 (BRCA1) C-terminal (BRCT) domain-containing protein, has been shown to play important roles in normal embryonic development, ribosome biogenesis, DNA replication, chromosomal stability and cell cycle progression^25–28^. Previous studies have demonstrated that PES1 is widely expressed in developing tissues, but is not observed in any adult tissues except for the ovary^26,27^. However, the subsequent studies have revealed that PES1 is over-expressed in some cancers such as stomach cancer^29^, prostatic cancer^30,31^, breast cancer^32,33^, head and neck squamous cell cancer^34^, colon cancer^35^, malignant astrocytomas and glioblastomas^36,37^ and ovarian cancer^38^. High PES1 expression is associated with the worse overall and relapse-free survival of patients with malignant tumor. The increased expression of PES1 transforms both mouse and human fibroblasts^39^, while the repression of PES1 inhibits the proliferation and tumorigenicity of breast cancer cells^32,33^. These data suggest that PES1 promotes the proliferation and malignant transformation of cells and may contribute to the occurrence and development of tumor.

Recently, Cheng *et al*.^33^ and Li *et al*.^38^ reported a novel function of PES1 that modulates the balance between ERα and ERβ protein levels through the ubiquitin-proteasome pathway, which contributes to the growth of breast and ovarian cancer cells. However, its role in connection with the balance between ERα and ERβ protein levels and the ERα/ERβ protein ratio in PTC has not been studied yet. Here, we examined PES1 protein level in human PTC tissues and cells and assessed the correlations of PES1 protein level with ERα and ERβ protein levels, with various clinicopathological features of PTC patients and with the ERα/ERβ protein ratio and the proliferation, invasion and migration of PTC cells.

## Results

### PES1 and ERα protein levels are significantly upregulated and ERβ protein level is significantly downregulated in PTC tissues

**Figure 1:**
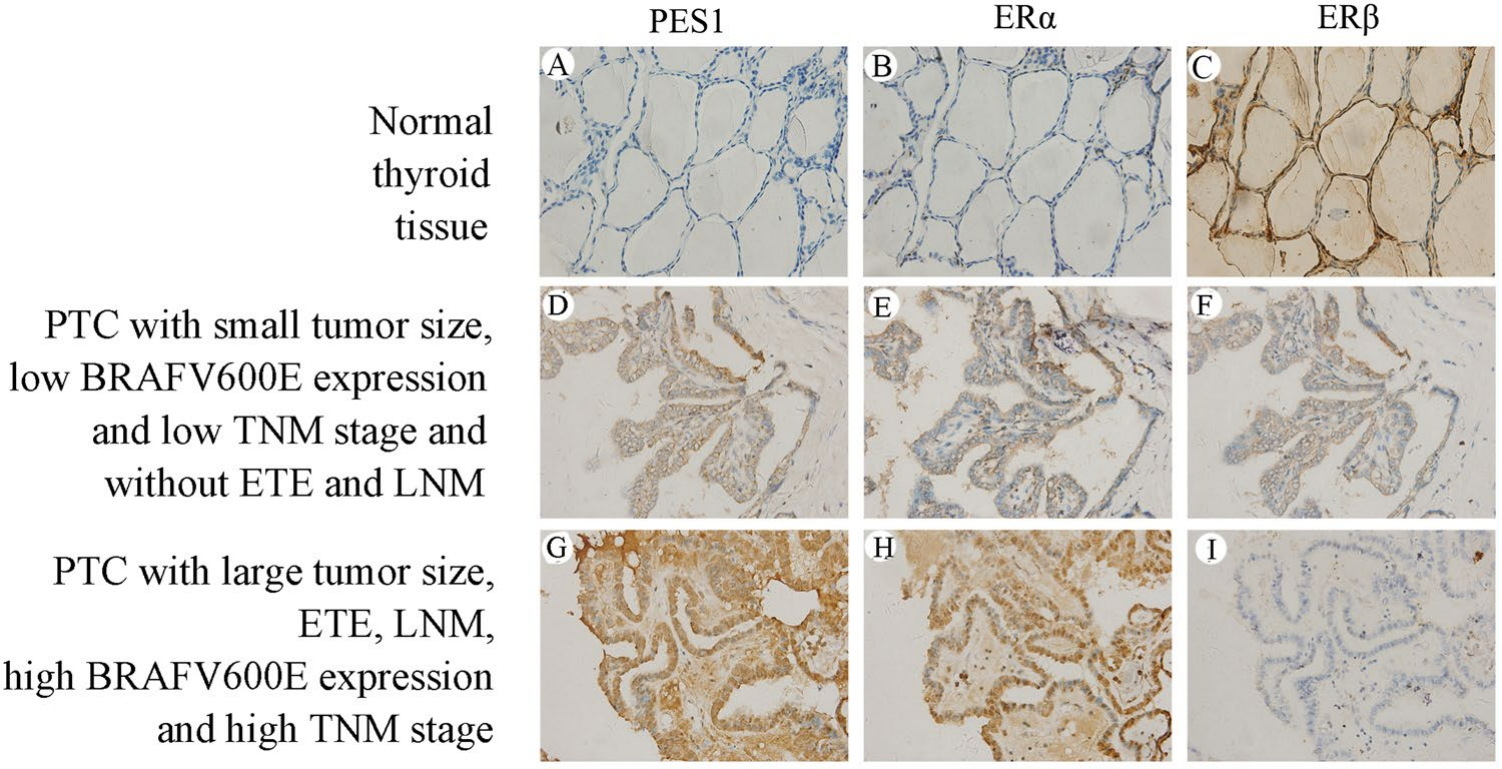
IHC staining of PES1, ERα and ERβ. The first row (**A–C**) is the IHC staining of an example of normal thyroid tissues, showing almost no follicular cells with staining for PES1 (**A**), a few of follicular cells with weak staining for ERα (**B**) and a lot of follicular cells with strong staining for ERβ (**C**). The second row (**D**–**F**) is the IHC staining of an example of PTC tissues with small tumor size, low BRAFV600E expression and TNM stage I and without ETE and LNM, showing quite a few of tumor cells with moderate staining for PES1 (**D**), ERα (**E**) and ERβ (**F**). The third row (**G**–**I**) is the IHC staining of an example of PTC tissues with large tumor size, ETE, LNM, high BRAFV600E expression and TNM stage IV, showing a lot of tumor cells with strong staining for PES1 (**G**) and ERα (**H**), however, a few of tumor cells with weak staining for ERβ (**I**).

PES1, ERα and ERβ protein levels in PTC and normal thyroid tissues were examined using immunohistochemical (IHC) staining and the examples of IHC staining for the three molecules were shown in Fig. 1A–C is an example of normal thyroid tissues showing almost no follicular cells with staining for PES1 (A) and a few of follicular cells with weak staining for ERα (B), however, a lot of follicular cells with strong staining for ERβ (C). D–F is an example of PTC tissues with small tumor size, low BRAFV600E expression and TNM stage I and without ETE and LNM, showing quite a few of tumor cells with moderate staining for PES1 (D), ERα (E) and ERβ (F). G–I is an example of PTC tissues with large tumor size, ETE, LNM, high BRAFV600E expression and TNM stage IV, showing a lot of tumor cells with strong staining for PES1 (G) and ERα (H), however, a few of tumor cells with weak staining for ERβ (I). It was indicated that PES1 and ERα protein levels are gradually increased and ERβ protein level is decreased by degree in the occurrence and development of PTC. As shown in Table 1, the majority of normal thyroid tissues were negative or had an IHC score 1 for PES1 and were negative or had an IHC score 1, 2, 3 or 4 for ERα, whereas none of cases showed high expression (IHC score ≥5) of PES1 and only 19 cases displayed high expression of ERα. On the other hand, however, only 6 normal thyroid tissues were negative for ERβ and the majority of cases had an IHC score 1–8, whereas 112 cases exhibited high expression of ERβ. High expression rates in normal thyroid tissues were 0%, 9.5% and 56% for PES1, ERα and ERβ, respectively. By contraries, the majority of the PTC tissues were positive and had an IHC score 1–8 for PES1 and ERα, whereas 104 and 103 of the PTC cases exhibited high expression (IHC score ≥5) of PES1and ERα, respectively. On the other hand, however, a significant number of PTC cases were negative for ERβ and only 30 PTC cases showed high expression of ERβ. High expression rates in PTC tissues were 52%, 51.5% and 15% for PES1, ERα and ERβ, respectively. It was indicated that PES1 and ERα protein levels are significantly upregulated and ERβ protein level is significantly downregulated in PTC tissues when compared with those in normal thyroid tissues (*P* < 0.001) (Table 2).

**Table 1:** The IHC scores of PES1, ERα and ERβ in 200 PTC and 200 normal thyroid tissues according to the scoring system.

Score	PES1		ERα		ERβ	
	Normal thyroid tissue *(n)*	PTC *(n)*	Normal thyroid tissue *(n)*	PTC *(n)*	Normal thyroid tissue *(n)*	PTC *(n)*
0						
Negative	176	11	69	9	6	71
+						
1	23	18	39	14	12	37
2	1	21	33	20	19	26
3	0	23	21	26	24	19
4	0	23	19	28	27	17
++						
6	0	25	12	30	30	18
8	0	27	6	39	41	12
+++						
9	0	35	1	22	27	0
12	0	17	0	12	14	0

The IHC scores in PTC and normal thyroid tissues were determined as the multiplication of proportion score and intensity score.

**Table 2:** Correlations of PES1, ERα and ERβ protein levels with clinicopathological characteristics in 200 PTC patients.

Characteristics	Case (*n*)	PES1			ERα			ERβ		
		Low	High	*P* value	Low	High	*P* value	Low	High	*P* value
Tissue type										
Normal thyroid tissue	200	200	0		181	19		88	112	
PTC	200	96	104	<0.001	97	103	<0.001	170	30	<0.001
Classic PTC	142	69	73	0.165	70	72	0.286	124	18	0.222
Follicular Variant of PTC	21	12	9		13	8		18	3	
Tall Cell Variant of PTC	24	7	17		8	16		17	7	
Oncocytic Variant of PTC	13	8	5		6	7		11	2	
Age (years)										
<45	24	14	10	0.280	15	9	0.143	18	6	0.144
≥45	176	82	94		82	94		152	24	
Gender										
Male	46	26	20	0.187	27	19	0.115	36	10	0.145
Female	154	70	84		70	84		134	20	
Tumor size (cm)										
T1 ≤ 2	62	39	23	0.005	41	21	0.003	46	16	0.007
2 < T2 ≤ 4	71	34	37		31	40		61	10	
T3 > 4	67	23	44		25	42		63	4	
Extrothyroid extension (ETE)										
Absent	161	86	75	0.002	87	74	0.001	131	30	0.003
Present	39	10	29		10	29		39	0	
Lymph node metastasis (LNM)										
Absent	110	63	47	0.004	63	47	0.006	86	24	0.003
Present	90	33	57		34	56		84	6	
BRAFV600E										
Low	67	41	26	0.008	43	24	0.002	50	17	0.004
High	133	55	78		54	79		120	13	
TNM stage										
I-II	100	59	41	0.002	60	40	0.001	77	23	0.002
III-IV	100	37	63		37	63		93	7	

*P*-values derived using Chi-square test to compare the protein levels of PES1, ERα and ERβ between subgroups defined by each clinicopathological parameter; *P* < 0.05 was considered to be statistically significant.

### High PES1 and ERα protein levels and low ERβ protein level are correlated with the aggressive behaviors of PTC patients

To explore whether and how PES1, ERα and ERβ protein levels are correlated with the clinicopathological features of PTC patients, we systematically assessed the correlations of PES1, ERα and ERβ protein levels with various clinicopathological characteristics of the PTC patients using Chi-square test. As shown in Table 2, there were no statistically significant correlations of PES1, ERα and ERβ protein levels with histologic subtype (*P* = 0.165, 0.286, 0.222), age (*P* = 0.280, 0.143, 0.144) and gender (*P* = 0.187, 0.115, 0.145) of PTC patients. However, PES1, ERα and ERβ protein levels were significantly correlated with tumor size (*P* = 0.005, 0.003, 0.007), ETE (*P* = 0.002, 0.001, 0.003), LNM (*P* = 0.004, 0.006, 0.003), BRAFV600E mutation (*P* = 0.008, 0.002, 0.004) and TNM stage (*P* = 0.002, 0.001, 0.002). PTC patients with large tumor size, ETE, LNM, high BRAFV600E expression and high TNM stage (III-IV) had higher rates of high PES1 and ERα protein expression and low ERβ protein expression. It was indicated that high PES1 and ERα protein levels and low ERβ protein level are correlated with the aggressive behaviors of PTC patients.

### PES1 protein level is positively correlated with ERα protein level and negatively correlated with ERβ protein level in PTC tissues

To explore whether and how PES1 protein level is correlated with ERα and ERβ protein levels, the correlations of PES1 protein level with ERα and ERβ protein levels in PTC tissues were assessed using Spearman rank test. As shown in Table 3, 81/200 cases showed high PES1 protein level associated with high ERα protein level. PES1 protein level was positively correlated with ERα protein level (*r*_*s*_ = 0.549, *P* < 0.001). Conversely, 101/200 cases displayed high PES1 protein level associated with low ERβ protein level. PES1 protein level was negatively correlated with ERβ protein level (*r*_*s*_ = −0.353, *P* < 0.001). In addition, high ERα protein level associated with low ERβ protein level was present in 98/200 cases. A significantly negative correlation (*r*_*s*_ = −0.293, *P* < 0.001) was also present between ERα and ERβ protein levels. It was indicated that PES1 protein level is positively correlated with ERα protein level and negatively correlated with ERβ protein level in PTC tissues.

**Table 3:** Correlations of PES1, ERα and ERβ protein levels between each other in 200 PTC tissues.

Protein	PES1				ERα			
	Low	High	*r*_*s*_	*P* value	Low	High	*r*_*s*_	*P* value
ERβ								
Low	69	101	−0.353	<0.001	72	98	−0.293	<0.001
High	27	3			25	5		
ERα								
Low	74	23	0.549	<0.001				
High	22	81						

*P*-values for Spearman rank test; PES1, ERα and ERβ were tested pairwise. *P* < 0.05 was considered to be statistically significant.

### PES1 protein level and ERα/ERβ protein ratio are upregulated in human PTC cells

**Figure 2:**
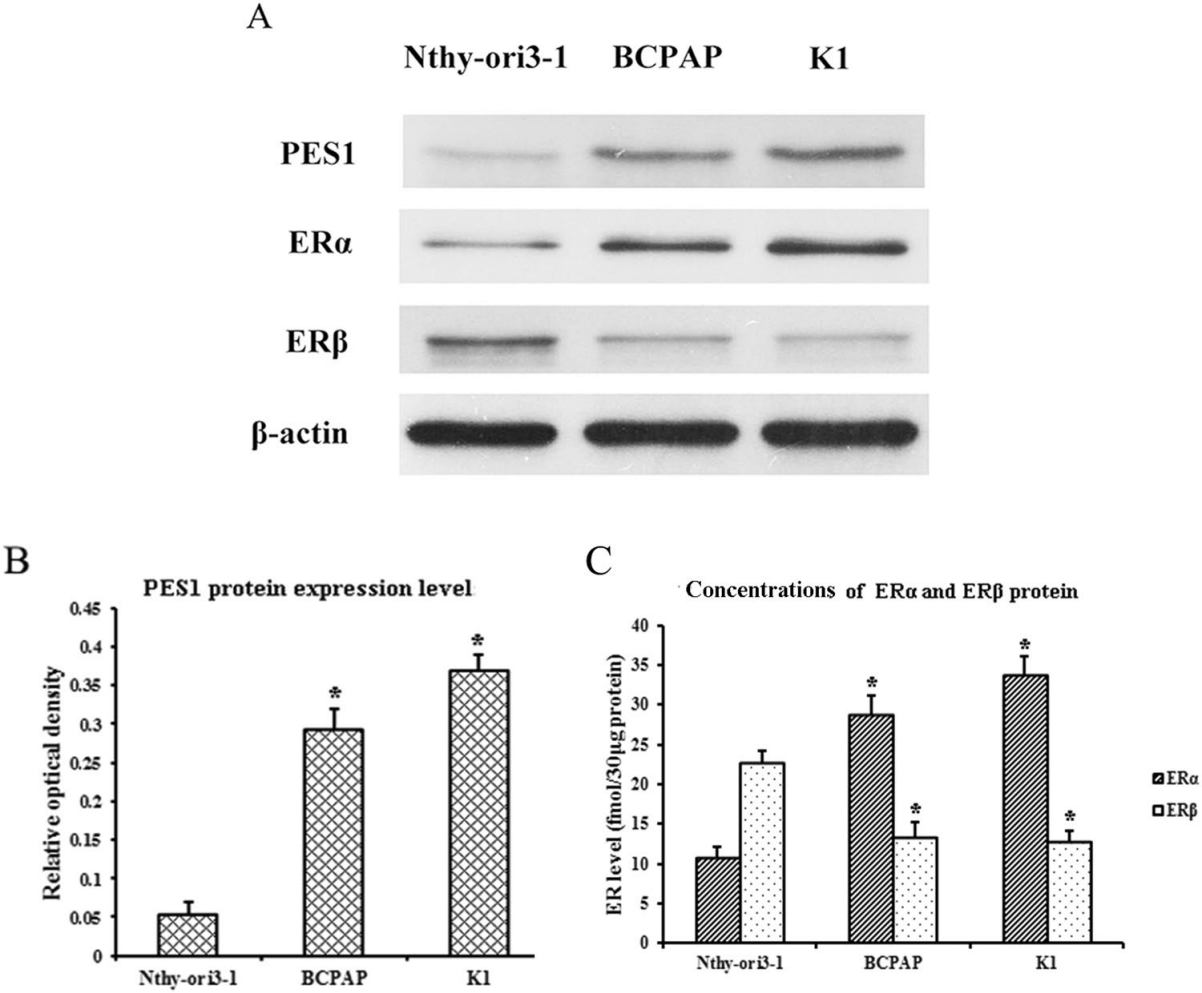
PES1 protein level and ERα/ERβ protein ratio in human PTC-derived BCPAP and K1 cells and normal thyroid-derived Nthy-ori3-1 cells. BCPAP, K1 and Nthy-ori3-1 cells were cultured, then the total protein of these cells was extracted and the protein levels of PES1, ERα and ERβ were assessed by Western blotting. β-actin served as an internal calibrator. (**A**) Blot examples of PES1, ERα and ERβ protein levels in BCPAP, K1 and Nthy-ori3-1 cells. (**B**) Bar diagrams of relative PES1 protein level in BCPAP, K1 and Nthy-ori3-1 cells. (**C**) The concentrations of ERα and ERβ protein in BCPAP, K1 and Nthy-ori3-1 cells. Data presented represent the mean of three independent experiments. Statistical differences between two groups were examined using Students t-test. *P < 0.05, compared with Nthy-ori3-1 normal thyroid cells.

To examine the PES1 protein level in human PTC and normal thyroid cells, we preformed Western blotting using human PTC-derived BCPAP and K1 cells with BRAFV600E mutation and normal thyroid-derived Nthy-ori3-1 cells without BRAFV600E mutation^40^. As shown in Fig. 2A,B, the PES1 protein level was much higher in BCPAP and K1 cells than that in Nthy-ori3-1 cells. To quantify the ERα/ERβ protein ratio in BCPAP, K1 and Nthy-ori3-1 cells, we measured the concentrations of ERα and ERβ protein in these cells by Western blotting as our previous method^41^. As shown in Fig. 2C, ERα and ERβ protein concentrations were 28.67 and 13.33 fmol/30 μg cell protein in BCPAP, 33.67 and 12.67 fmol/30 μg cell protein in K1 and 10.67 and 22.67 fmol/30 μg cell protein in Nthy-ori3-1 cells. The ERα/ERβ protein ratio was about 2.15:1 in BCPAP, 2.66:1 in K1 and 1:2.12 in Nthy-ori3-1cells, respectively. It was indicated that the PES1 protein level and the ERα/ERβ protein ratio are upregulated in human PTC cells when compared with those in normal thyroid cells.

### ERα promotes and ERβ inhibits the proliferation, invasion and migration of human PTC cells and normal thyroid cells

**Figure 3:**
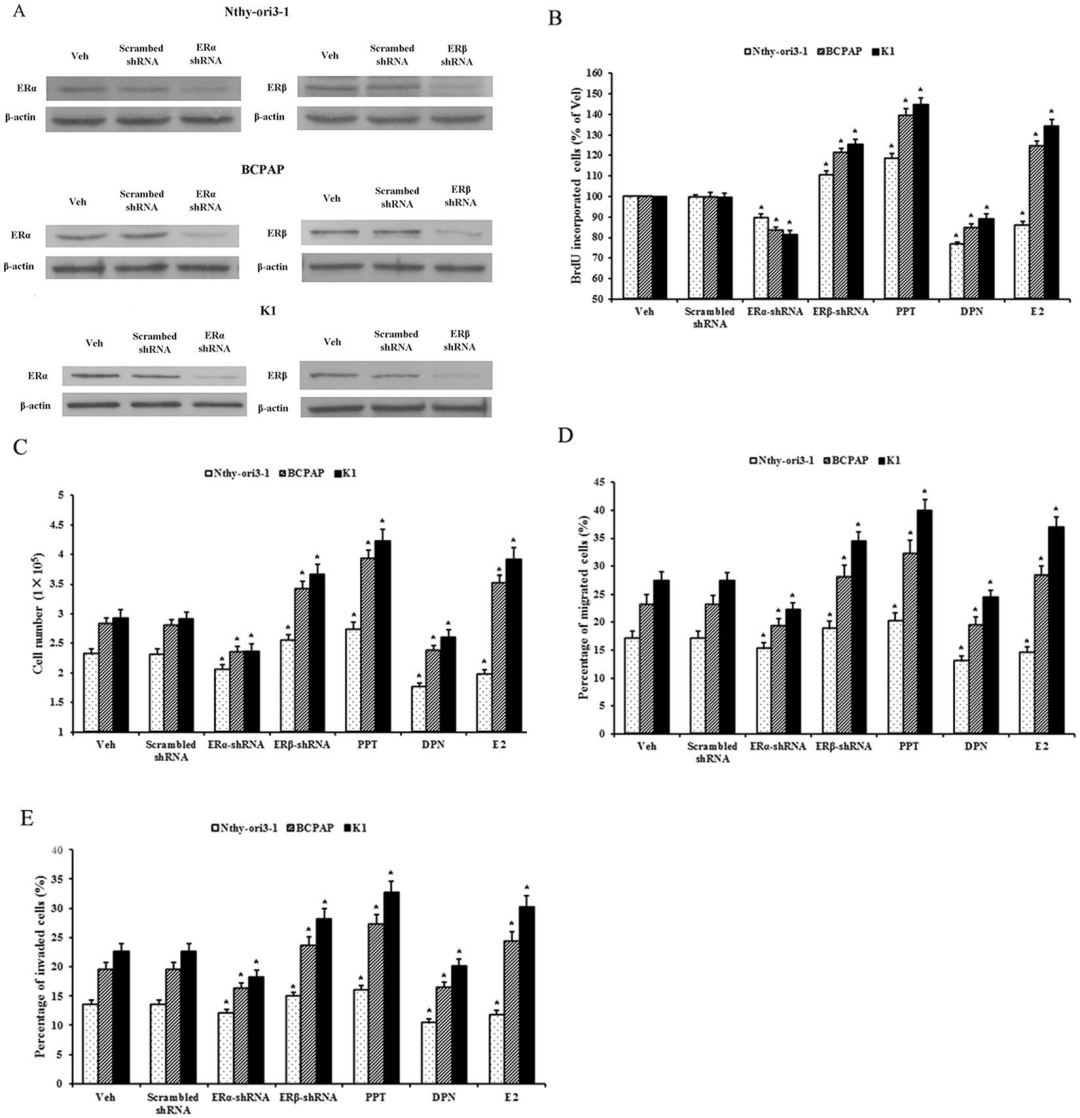
ERα promotes and ERβ inhibits the proliferation, invasion and migration of human PTC cells and normal thyroid cells. BCPAP, K1 and Nthy-ori3-1 cells were stably transfected with the expression vectors of ERα-shRNA, ERβ-shRNA and scrambled shRNA or were exposed to 10 nM of E2, PPT and DPN for 72 h, respectively. (**A**) Knockdown of ERα and ERβ protein levels by ERα-shRNA and ERβ-shRNA. (**B** and **C**) The proliferation of these cells was assessed by BrdU incorporation and cell count assays. (**D** and **F**) The migration and invasion of these cells were evaluated by Transwell assay. Data presented represent the mean of three independent experiments. Statistical differences between two groups were examined using Students t-test. *P < 0.05, compared with non treatment (Veh).

To explore the effects of ERα and ERβ on the proliferation, invasion and migration of human PTC and normal thyroid cells, ERα-shRNA and ERβ-shRNA expression vectors were used to generate the stable transfected cells with knockdown of ERα and ERβ. Meanwhile, PPT (ERα-selective agonist) and DPN (ERβ-selective agonist) were used to stimulate ERα and ERβ, respectively. As shown in Fig. 3, ERα-shRNA and ERβ-shRNA expression vectors effectively silenced ERα and ERβ expression, respectively, in BCPAP, K1 and Nthy-ori3-1cells. Compared with scrambed shRNA, ERα-shRNA decreased and ERβ-shRNA increased the proliferation, invasion and migration of human PTC-derived BCPAP and K1 cells and normal thyroid-derived Nthy-ori3-1 cells. Compared with the control (vehicle, Veh), ERα agonist PPT increased and ERβ agonist DPN decreased the proliferation, invasion and migration of these cells. It was indicated that ERα promotes and ERβ inhibits the proliferation, invasion and migration of human PTC cells and normal thyroid cells. Interestingly, E2 increased the proliferation, invasion and migration of human PTC-derived BCPAP and K1 cells with an ERα/ERβ protein ratio of >1, however, decreased the proliferation, invasion and migration of normal thyroid-derived Nthy-ori3-1 cells with an ERα/ERβ protein ratio of <1. It was indicated that the balance between ERα and ERβ protein levels and the ERα/ERβ protein ratio are crucial to the effects of E2 on the proliferation, invasion and migration of these thyroid cells.

### PES1 upregulates the ERα/ERβ protein ratio and promotes the proliferation, invasion and migration of human PTC cells and normal thyroid cells

**Figure 4:**
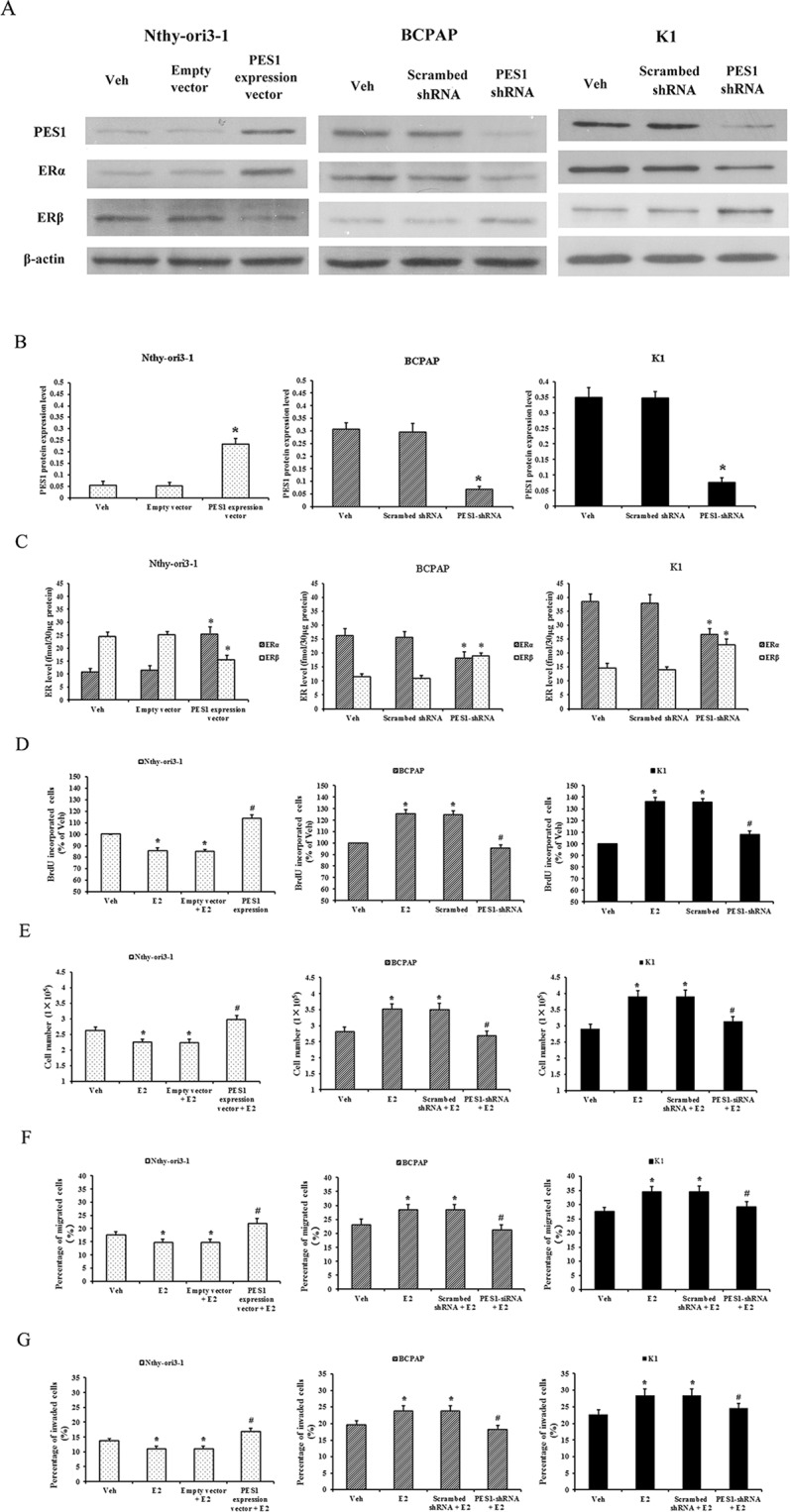
Effects of PES1 on the ERα/ERβ protein ratio and the proliferation, invasion and migration of human PTC and normal thyroid cells. The normal thyroid-derived Nthy-ori3-1 cells were stably transfected with the PES1 expression vector or empty vector and the PTC-derived BCPAP and K1 cells were stably transfected with the expression vector of PES1-shRNA or scrambed shRNA. The protein levels of PES1, ERα and ERβ in these stable transfected cells were assessed by Western blotting. β-actin served as an internal calibrator. The proliferation, invasion and migration of these stable transfected cells were assayed after exposure to E2 for 72 h. (**A**) Blot examples of PES1, ERα and ERβ protein levels in these stable transfected cells. (**B**) Bar diagrams of relative PES1 protein level in these stable transfected cells. (**C**) The concentrations of ERα and ERβ protein in these stable transfected cells. (**D** and **E**) The proliferation of these stable transfected cells was assessed by BrdU incorporation and cell count assays. (**F** and **G**) The migration and invasion of these stable transfected cells were assessed by Transwell assay. Data presented represent the mean of three independent experiments. Statistical differences between two groups were examined using Students t-test. *P < 0.05, compared with non treatment (Veh). ^#^*P* < 0.05, compared with E2 treatment alone.

To explore the effects of PES1 on the ERα/ERβ protein ratio and the proliferation, invasion and migration of human PTC cells and normal thyroid cells, PES1-shRNA expression vector was used to generate the stable transfected PTC cells with knockdown of PES1 and PES1expression vector was used to generate the stable transfected normal thyroid cells with increased PES1 expression. As shown in Fig. 4, compared with scrambed shRNA, PES1-shRNA reduced the ERα protein level and simultaneously elevated the ERβ protein level, and then resulted in a decrease of the ERα/ERβ protein ratio and a decrease of the promotion effects of E2 on the proliferation, invasion and migration of human PTC-derived BCPAP and K1 cells. Conversely, compared with empty vector, PES1 expression vector elevated the ERα protein level and simultaneously reduced the ERβ protein level, and then resulted in an increase of the ERα/ERβ protein ratio and an inhibition-promotion transition of the effects of E2 on the proliferation, invasion and migration in normal thyroid-derived Nthy-ori3-1 cells. It was indicated that PES1 elevates the ERα protein level and simultaneously reduces the ERβ protein level, i.e., upregulates the ERα/ERβ protein ratio, and then promotes the proliferation, invasion and migration of human PTC cells and normal thyroid cells.

## Discussion

Studies have shown that estrogen may be involved in the occurrence and development of PTC^1–5^, as has been shown in breast, endometrial and ovarian cancer^6^. Estrogen exerts its effects mainly via ERα and ERβ^7,8^. ERα and ERβ are often co-expressed and contribute to the physiological and pathophysiological responses of estrogen^42,43^. In this study, we detected ERα and ERβ protein levels in PTC tissues and cells using IHC staining and Western blotting, respectively. Compared with normal thyroid tissues and cells, the ERα protein level was upregulated and the ERβ protein level was downregulated in PTC tissues and cells. PTC tissues had higher rates of high ERα and low ERβ protein expression. PTC cells had more than two times higher protein level of ERα than that of ERβ. This result is in line with the previous data showing that ERα and ERβ are co-expressed in normal and tumor tissues of the thyroid^20,21^, compared with normal thyroid tissues, the level of ERα is relatively higher than that of ERβ in PTC tissues^21–24^.

Although ERα and ERβ are architecturally similar with three functional domains of N-terminal domain (NTD), DNA binding domain (DBD) and ligand binding domain (LBD), there are obvious differences in their structures of NTD and LBD. The differences in their structures suggest that ERα and ERβ may have different functions^9^. Cell-based assays have shown that ERβ is generally less active than ERα and may influence ERα activity. Despite a small number of data associating ERβ with pro-growth and pro-survival when present alone in ERα-negative estrogen related cancer tissues and cells, a large number of data both *in vitro* and *in vivo* support that ERβ acts as an anti-proliferative and pro-apoptotic factor, especially when co-expressed with ERα^44,45^. Most studies have revealed that ERα promotes cell proliferation, invasion and migration and has been shown to have tumor-promoting effects, whereas ERβ, when co-expressed with ERα, may play an inhibitory role against the ERα-mediated tumor-promoting effects^14–16^. Thus, the complement of ER isoform could influence the biological responses and the balance and ratio between ERα and ERβ protein levels would be critical in defining the overall response^14–18^. In this study, we showed that compared with normal thyroid tissues, the ERα protein level was upregulated and the ERβ protein level was downregulated in PTC tissues. The ERα protein level was positively correlated with the aggressive behaviors of PTC patients, conversely, the ERβ protein level was negatively correlated with the aggressive behaviors of PTC patients. PTC patients with large tumor size, ETE, LNM, high BRAFV600E expression and high TNM stage (III-IV) had higher rates of high ERα and low ERβ protein expression. Moreover, cell-based assays showed that the ERα/ERβ protein ratio was greater than one (>1) in human PTC-derived BCPAP and K1 cells with the BRAFV600E mutation, conversely, smaller than one (<1) in normal thyroid-derived Nthy-ori3-1 cells without the BRAFV600E mutation. ERα promoted and ERβ inhibited the proliferation, invasion and migration of these PTC cells and normal thyroid cells. Interestingly, E2 increased the proliferation, invasion and migration of human PTC cells with an ERα/ERβ protein ratio of >1, however, decreased the proliferation, invasion and migration of normal thyroid cells with an ERα/ERβ protein ratio of <1. It was indicated that the ERα/ERβ protein ratio is crucial to the effects of E2 on the proliferation, invasion and migration of these thyroid cells.

PES1, a BRCT domain-containing protein, is essential for ribosome biogenesis, nucleologenesis and cell growth, which are all important components that determine the cell proliferation rate^25,28^. The increased expression of PES1 is involved in the proliferation and malignant conversion of cells and may contribute to the occurrence and development of some human cancers such as prostate, head and neck, stomach, colon, breast and ovarian cancer^29–39^. Recently, Cheng *et al*.^33^ and Li *et al*.^38^ reported a novel function of PES1 that regulates the balance between ERα and ERβ protein levels. They found that PES1 enhances the stability of ERα while simultaneously targeting ERβ for proteasome degradation, thereby increasing the protein level of ERα and decreasing that of ERβ, which contributes to the occurrence and development of breast cancer and ovarian cancer. As some ERs regulators have been found to have tissue specificity that modulate their activities^46,47^, in this study, we examined PES1 protein level in PTC tissues and cells and assessed the correlations of its protein level with the ERα and ERβ protein levels and with the occurrence and development of PTC. We found that PES1 protein level was positively correlated with ERα protein level and negatively correlated with ERβ protein level in human PTC tissues and cells. Compared with normal thyroid tissues, PES1 protein level was significantly increased in PTC tissues and was associated with the aggressive behaviors of PTC patients. PTC patients with large tumor size, ETE, LNM, high BRAFV600E expression and high TNM stage (III-IV) had higher rates of high PES1 and ERα protein levels and low ERβ protein level. Increased PES1 and ERα protein levels and decreased ERβ protein level were associated with the occurrence and development of PTC. Cell-based assays showed that PES1 elevated ERα protein level and simultaneously reduced ERβ protein level and resulted in an upregulated ERα/ERβ protein ratio, and then promoted the proliferation, invasion and migration of human PTC cells.

In summary, ERα and ERβ were co-expressed in human PTC tissues and cells. ERα promoted and ERβ inhibited the proliferation, invasion and migration of PTC cells. PES1 modulated the balance between ERα and ERβ by elevating the ERα protein level and simultaneously reducing the ERβ protein level, then upregulated the ERα/ERβ ratio and promoted the proliferation, invasion and migration of PTC cells. In PTC tissues, PES1 protein level was positively correlated with ERα protein level and negatively correlated with ERβ protein level. PES1 and ERα protein levels were gradually increased and ERβ protein level was decreased by degree in the occurrence and development of PTC. Increased PES1 and ERα protein levels and decreased ERβ protein level were correlated with the aggressive behaviors of PTC patients such as large tumor size, ETE, LNM, high BRAFV600E expression and high TNM stage. It was suggested that PES1 promotes the occurrence and development of PTC by elevating the ERα protein level and reducing the ERβ protein level, and then upregulating the ERα/ERβ protein ratio.

## Materials and Methods

### Case selection and tissue samples

Tumor tissue samples were obtained from 200 PTC patients who underwent initial thyroidectomy in the Department of Surgery, the First Affiliated Hospital, Chongqing Medical University, between January 2015 and January 2017. At initial thyroidectomy, cervical lymph node dissection (CLND) was performed, tumor size was assessed, histologic subtype was defined and extrathyroidal extension (ETE) and BRAFV600E mutation were evaluated. Of the 200 cases, 142 were classic PTC, 21 follicular variant of PTC, 24 tall cell variant of PTC and 13 oncocytic variant of PTC. There were 39 patients with ETE, 90 patients with lymph node metastasis (LNM), 133 patients with high BRAFV600E expression, 62 patients with tumor size of ≤2 cm, 71 patients with tumor size of >2 and ≤4 cm and 67 patients with tumor size of >4 cm. Of this patient cohort, 46 were men and 154 women; 24 patients were aged <45 years and 176 were aged ≥45 years. According to the seventh edition of thyroid cancer tumor-node-metastasis (TNM) staging system by American Joint Committee on Cancer, 50 patients were stage I, 50 patients stage II, 50 patients stage III and 50 patients stage IV. For statistical analysis, stage I and II were combined into low TNM stage (I-II) and stage III and IV were combined into high TNM stage (III-IV). Besides, 200 normal thyroid tissues were taken from the contralateral lobes of PTC tissues. The study protocol was approved by the Ethics Committee of Chongqing Medical University and informed consent was obtained from all of the patients.

### Tissue microarray and IHC staining

Tissue microarray construction and IHC staining were performed as described previously^48^. Rabbit polyclonal anti-PES1 (ab72539), mouse monoclonal anti-ERα (ab1104) and mouse monoclonal anti-ERβ (ab1103) were purchased from Abcam (Abcam, Cambridge, MA, USA). Mouse monoclonal anti-BRAFV600E (26039) was obtained from NewEast Bioscience (NewEast Bioscience, Malvern, PA, USA). These antibodies were used as primary antibodies at 1:50 dilution. Biotinylated goat-anti-rabbit and goat-anti-mouse IgG (ZB-2010 and ZB-2305, Zhongshan Golden Bridge Biotechnology, China) were used as secondary antibodies at 1:500 dilution.

### IHC scoring

A semi-quantitative IHC scoring assessment was performed by two observers blinded to the diagnosis. IHC score was assigned based on the percentage of positive cells and the staining intensity. The percentage was evaluated and assigned a score of 0−4: 0, <5% positive cells; 1, 6–25% positive cells; 2, 26–50% positive cells; 3, 51–75% positive cells; and 4, >75% positive cells. The staining intensity was evaluated and assigned a score of 0−3: 0, no staining; 1, weak staining; 2, moderate staining; and 3, strong staining. The IHC score was then assigned to each sample by multiplying the percentage score and the staining intensity score, and thus the score ranged from 0 to 12. For statistical analysis, a final IHC score of 0 (negative) or 1–4 (+) was defined as low expression and a final IHC score of 5–8 (++) or 9–12 (+++) as high expression.

### Cell culture and treatment

Human PTC-derived BCPAP and K1 cells with BRAFV600E mutation and normal thyroid-derived Nthy-ori3-1 cells without BRAFV600E mutation^40^ were obtained from the American Type Culture Collection (ATCC, Manassas, VA, USA) and maintained in RPMI-1640 medium supplemented with 10% charcoal-stripped fetal bovine serum (FBS) (GBICO Co., Ltd., Grand Island, NY, USA). The cells were incubated in phenol-red free and serum free medium for 48 h before stimulation with 17β-estradiol (E2), propyl-pyrazole-triol (PPT) and diarylpropionitrile (DPN) (Sigma, St Louis, MO, USA). Cell treatments were performed as indicated in the respective figure legends.

### Construction of shRNA expression vectors for PES1, ERα and ERβ

For a vector-based RNA interference (RNAi) approach, short hairpin RNA (shRNA) was cloned into the BamHI-HindIII sites of the pRNAT-U6.1/neo vector (GenScript Corp. Piscataway, NJ, USA). RNAi target sequences were selected from the human PES1, ERα and ERβ sequences (GenBank accession NM_001282328.1 for PES1, NM_001122741.1 for ERα and NM_001291723.1 for ERβ). The candidate target sequences were analyzed by BLAST search to ensure that the knockdown would be unique to PES1, ERα and ERβ. The specific shRNA sequences were 5′-*GGATTC*CCGGCCAGAAGATCATGTTTGGCAATTCAAGAGATTGCCAAACATGATCTTCTGG**TTTTTG***AAGCTT*-3′ for PES1, 5′-*GGATTC*CCGGCTACAGGCCAAATTCAGATAA TTCAAGAGATTATCTGAATTTGGCCTGTAG**TTTTTG***AAGCTT*-3′ for ERα and 5′-*GGATTC* CCGGGCGAGTAACAAGGGCATGGAATTCAAGAGATTCCATGCCCTTGTTACTCGC**TTTTTG***AAGCTT*-3′ for ERβ. A nonsilencing RNAi control vector comprising a scrambled shRNA sequence was generated with the following oligonucleotide 5′-*GGATTC*CCGG**CC**TAAGGTTAAGTCGCCC TCGTTCAAGAGACGAGGGCGACTTAACCTTAGG**TTTTTG***AAGCTT*-3′. The underlined, boldface and italic letters denote the hairpin loop, terminal signal and target sites of BamHI and HindIII restriction enzymes, respectively. These shRNA sequences were digested with BamHI and HindIII restriction enzymes (Takara Biotechnology Co., Ltd., Dalian, China) and cloned into the pRNAT-U6.1/neo vector with T4 ligase (Takara). The resulting constructs were verified by direct sequencing (Sangon Biotech Co., Ltd., Shanghai, China).

### Construction of PES1 expression vector

Full-length PES1 cDNA was amplified by PCR using human universal QUICK clone cDNA (Clonetech Laboratories Inc., Mountain View, CA, USA) as a PCR template and specific primers based on PES1 cDNA sequence (GenBank Accession: NC_000022.11). The specific primers were as follows: forward 5′-*ATAATT*GGATTCGCCACCATGGGAGGCCTTGAGAAGA-3′ and reverse 5′-*ATTATA*AAGCTTCTCCGGCCTTGCCTTCTTGGCCTTC-3′. The underlined and italic letters denote the target sites of BamHI and HindIII restriction enzymes and the additional sequence, respectively. The PCR product containing the PES1 open-reading frame sequence was digested with BamHI and HindIII restriction enzymes (Takara) and cloned into the pcDNA3.1 expression vector (Invitrogen, Carlsbad, CA, USA) with T4 ligase (Takara). The resulting construct was verified by direct sequencing (Sangon Biotech Co., Ltd, Shanghai, China).

### Transfection

A single day prior to transfection, cells were seeded in 6-well culture plates at a density of 5.0 × 10^5^ cells per well (2.0 ml/well). Transfection was performed using Lipofectamine 2000 reagent (Invitrogen) in accordance with the standard protocol of the manufacturer. In brief, transfection was initiated when the cells were 70–80% confluent. For each well, 5 μg of plasmid DNA was added into 250 μl of Opti-MEM (Invitrogen), 5 μl of lipofectamine 2000 into 250 μl of Opti-MEM, and then mixed plasmid DNA with Lipofectamine 2000. The mixture was added to cells in the 6-well culture plates, giving an end volume of 1 ml. The Opti-MEM medium containing complex was incubated for 8 h at 37 °C, then replaced with 2 ml of standard growth media and cultured for 48 h at 37 °C. Stable transfected cells were selected in the presence of 400 μg/ml of G418 (GIBCO) for 3 weeks with medium change on every 4th or 5th day.

### Protein extraction and Western blotting

Cells were harvested and gently lysed for 30 min in 5 volumes of ice-cold RIPA buffer (Sigma) supplemented with Complete Protease Inhibitor Cocktail (Roche Diagnostics, Mannheim, Germany) at a ratio of 1000:1. Cell lysates were centrifuged at 10 000 × g for 10 min at 4 °C to obtain the supernatants. The protein content of cell lysates was quantified by the Bio-Rad Dc Protein Assay kit (Bio-Rad Laboratories, Hercules, CA, USA). Western blotting was performed as described previously. Briefly, 30 μg of total cell protein was separated by sodium dodecyl sulfate-polyacrylamide gel electrophoresis (SDS-PAGE) and transferred to polyvinylidene fluoride (PVDF) membranes (Millipore, Billerica, MA, USA). The transferred membranes were probed with anti-PES1 (ab72539, Abcam), anti-ERα (ab1104, Abcam), anti-ERβ (ab1103, Abcam) and anti-β-actin (ab8227, Abcam) primary antibodies overnight at 4 °C and at 1:500 dilution. Subsequently, the membranes were washed and reacted with peroxidase-conjugated goat anti-mouse or goat anti-rabbit secondary antibodies (sc-2005 and sc-2004, Santa Cruz Biotechnology, CA, USA) for 1 h at room temperature and at 1:2000 dilution. The immunoreactive bands were visualized using an enhanced chemioluminescent ECL reagent (Amersham Biosciences, Piscataway, NJ, USA) and quantified using the TINA version 2.09 program package by normalizing to β-actin signal.

### BrdU assay

Cells were seeded in 96-well culture plates at a density of 5 × 10^3^ cells per well, incubated for 24 h, serum-deprived for 48 h and treated as indicated in the figure legends. Then a 5-bromo-2′-deoxyuridine (BrdU) incorporation colorimetric ELISA kit (Roche Diagnostics) was used to assay the cell proliferation in accordance with the standard protocol of the manufacturer.

### Cell count assay

Cells were seeded in 6-well culture plates at a density of 2 × 10^5^ cells per well, incubated for 24 h, serum-deprived for 48 h and treated as indicated in the figure legends. Then cells were harvested using Trypsin-EDTA, resuspended in the fresh medium and diluted in 0.4% trypan blue at a volume ratio of 1:1. The cell number was manually counted using a haemocytometer under microscope.

### *In vitro* migration and invasion assays

*In vitro* migration and invasion assays were performed using transwell chambers (8-μm pore size, 24-well insert, Corning Inc., Corning, NY, USA) according to the manufacturer’s instructions. Briefly, 3 × 10^4^ serum-starved cells were resuspended and seeded in the upper chambers in phenol-red free and serum-free medium supplemented with or without E2, PPT or DPN. The lower chambers were filled with phenol-red free medium supplemented with 10% charcoal-stripped FBS as a chemoattractant. For the invasion assay, the inserts were pre-coated with extracellular matrix gel (BD Biosciences, Bedford, MA, USA). Following 72 h incubation, MTT solution (0.5 mg/mL) was added to the upper and lower chambers and incubated for another 4 h. After this, the cells in the upper membrane surface (residual cells) and the cells in the lower membrane surface (migrated or invaded cells) were scraped off with a cotton swab and dissolved in 400 μl of DMSO, respectively. Then 100 μl of the dissolved solution was taken and the absorbance was measured at 570 nm. Data were expressed as a percentage of migrated and invaded cells, i.e., A/(A + B) × 100, where A is the absorbance of the migrated or invaded cells and B the absorbance of the residual cells.

### Statistics

Statistical analyses were performed using the SPSS version 18.0 (SPSS Inc., Chicago, IL, USA). Data are presented as percentages and mean plus standard deviation, according to distribution. Significance was assessed using Chi-square, Spearman rank and Student’s t-tests, as appropriate, to compare groups. A *P*-value of < 0.05 was considered to be statistically significant.

### Statements for Materials and Methods

The study protocol was approved by the Ethics Committee of Chongqing Medical University and informed consent was obtained from all of the patients. All experiments were performed in accordance with the relevant guidelines and regulations.

## Data Availability

All data generated or analyzed during this study are included in this article (and its Supplementary Information files).
